# Supplementary material for: Phosphorylation‐dependent activation of the bHLH transcription factor ICE1/SCRM promotes polarization of the Arabidopsis zygote
Source: New Phytol. 2024 Nov 14;245(3):1029–39. doi: 10.1111/nph.20265 (PMC11711944; doi:10.1111/nph.20265)
Supplement: Supplementary file 1 — Fig. S1 Embryonic expression profiles and mutant alleles. Fig. S2 SCRM and WRKY2 act synergistically in promoting WOX8 expression. Fig. S3 Phenotypic analysis of scrm and scrm2 mutant combinations. Fig. S4 gWOX8Δ‐YFP expression is reduced in scrm and wrky2 mutants. Fig. S5 Stomatal and embryonic phenotypes in transgenic SCRM variants. Fig. S6 Gain‐of‐function defects of constitutively active YDA are partially suppressed in scrm‐D mutants. Table S1 Genotyping primer list. Table S2 Cloning and mutagenesis primer list. Please note: Wiley is not responsible for the content or functionality of any Supporting Information supplied by the authors. Any queries (other than missing material) should be directed to the New Phytologist Central Office. [file NPH-245-1029-s001.pdf]

New Phytologist

***New Phytologist* Supporting Information**

Article title: **Phosphorylation-Dependent Activation of the bHLH Transcription Factor ICE1/SCRM Promotes Polarization of the Arabidopsis Zygote**

Authors: Houming Chen, Feng Xiong, Alexa-Maria Wangler, Torren Bischoff, Kai Wang, Yingjing Miao, Daniel Slane, Rebecca Schwab, Thomas Laux, Martin Bayer

Article acceptance date: 24 October 2024

The following Supporting Information is available for this article:

Fig. S1 Embryonic expression profiles and mutant alleles.

Fig. S2 SCRM and WRKY2 act synergistically in promoting *WOX8* expression.

Fig. S3 Phenotypic analysis of *scrm* and *scrm2* mutant combinations.

Fig. S4 *gWOX8Δ-YFP* expression is reduced in *scrm* and *wrky2* mutants.

Fig. S5 Stomatal and embryonic phenotypes in transgenic *SCRM* variants

Fig. S6 Gain-of-function defects of constitutively active YDA are partially suppressed in *scrm-D* mutants

Table S1 Genotyping primer list

Table S2 Cloning and mutagenesis primer list

Fig. S1 Embryonic expression profiles and mutant alleles. (a) Publicly available RNA-seq expression profiles in the early embryo for *SCRM* and *SCRM2* as reported in Zhao *et al*., 2019. (b) Schematic depiction of CRISPR/Cas9-mediated *ssp-6* and *scrm-cr* mutant alleles. (c) Boxplot diagram of zygote lengths. The sample size is given above the x axis, genotype below. Center lines show the medians; box limits indicate the 25th and 75th percentiles; whiskers extend 1.5 times the interquartile range from the 25th and 75th percentiles; red crosses represent sample means; data points are plotted as gray dots. Letters above boxes refer to individual groups in a one-way ANOVA with a post hoc Tukey test ($p < 0.05$).

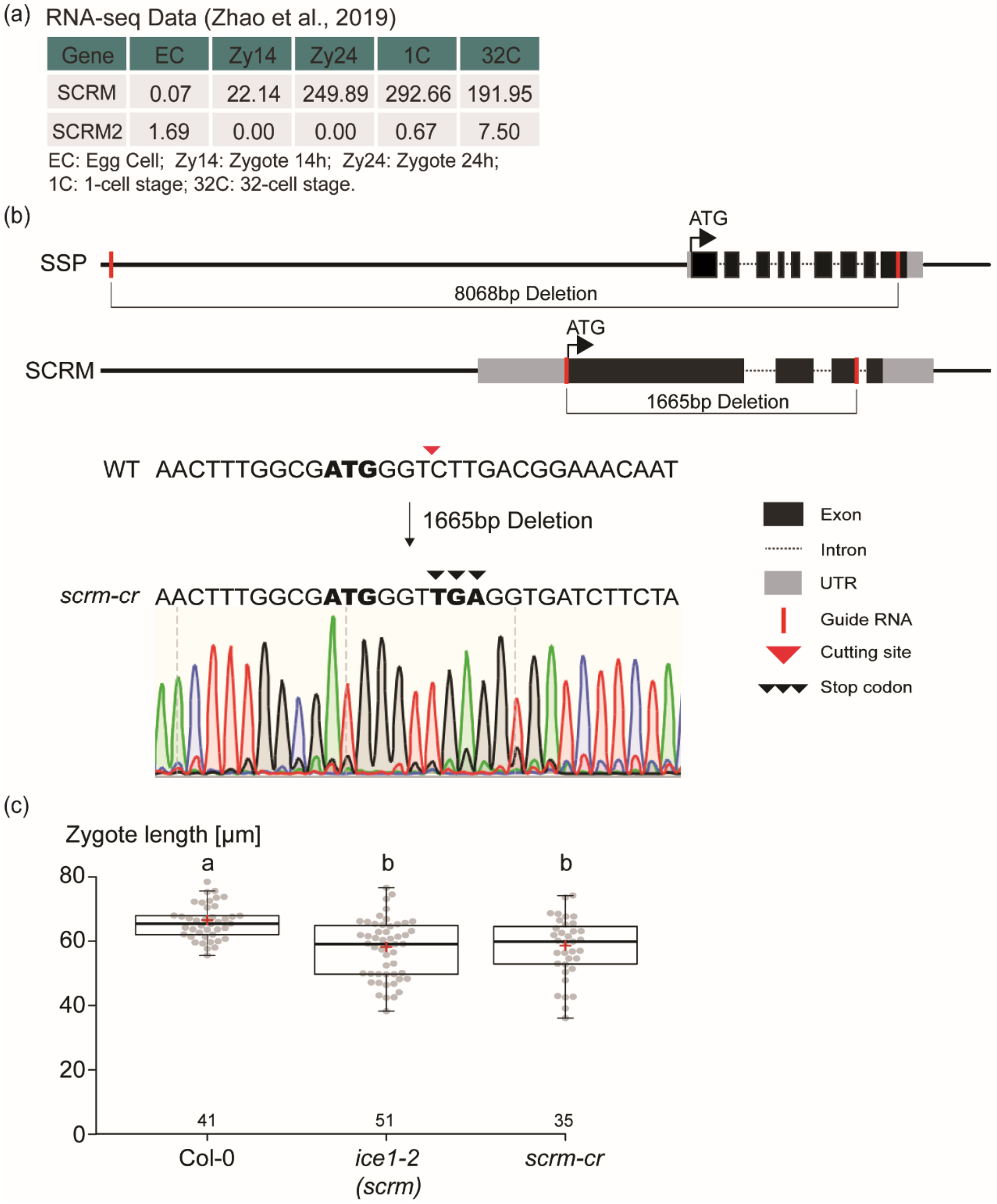

Fig. S2 SCRM and WRKY2 act synergistically in promoting *WOX8* expression. (a) DIC microscopy images of transition stage embryos. Genotypes are given below figure panels. Scale bars, 20 µm. (b) Boxplot diagram of suspensor lengths at transition stage. The sample size is given above the x axis, the genotype is given below. Center lines show the medians; box limits indicate the 25th and 75th percentiles; whiskers extend 1.5 times the interquartile range from the 25th and 75th percentiles; red crosses represent sample means; data points are plotted as gray dots. Letters above boxes refer to individual groups in a one-way ANOVA with a post hoc Tukey test ($p < 0.05$). (c) Split-Luciferase assay to test protein interaction. Boxplots of 8 replicates for each protein combination. Center lines show the medians; box limits indicate the 25th and 75th percentiles; whiskers extend 1.5 times the interquartile range from the 25th and 75th percentiles; data points are plotted as gray dots. Protein expression was confirmed by Western Blot (d). (e) Dual luciferase activity assay in protoplasts of suspension cell cultures. Data is illustrated as means ± SD of three independent experimental replicates, asterisk indicates statistical differences in Student's t-test ($p<0.05$).

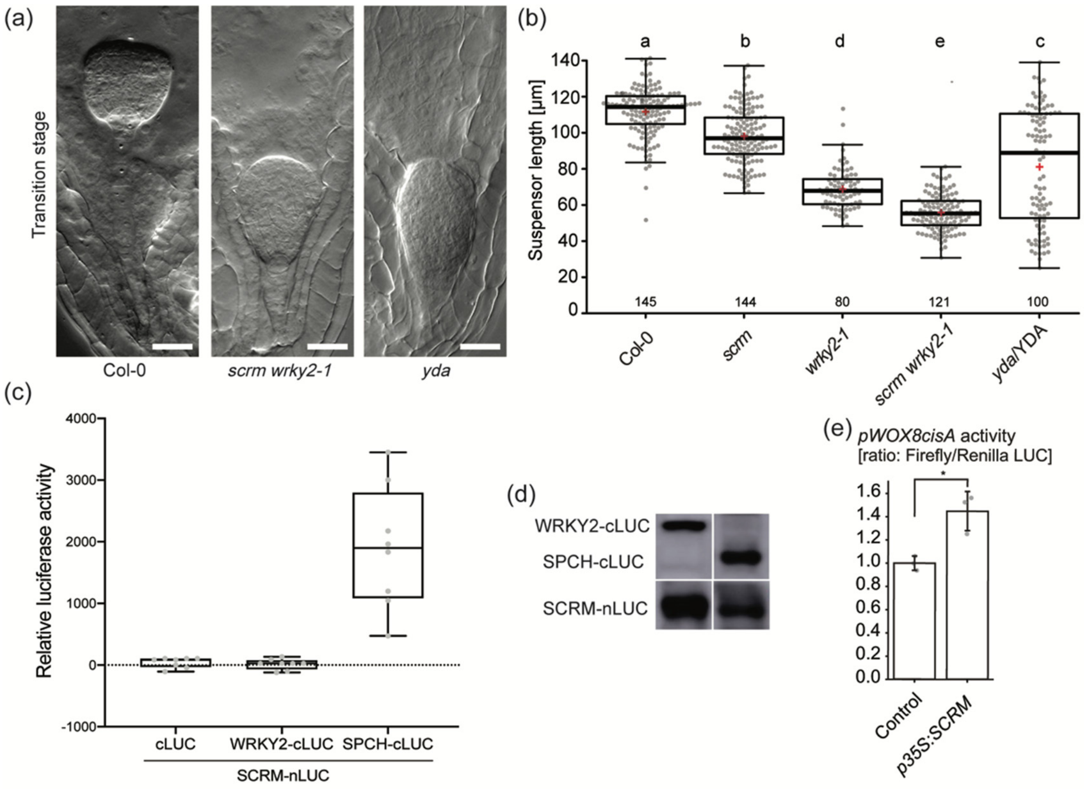

Fig. S3 Phenotypic analysis of *scrm* and *scrm2* mutant combinations. (a-c) Boxplot diagram of zygote lengths (a), zygote polarity (b) and suspensor length at transition stage (c). The sample size is given above the x axis, the genotype is given below. Center lines show the medians; box limits indicate the 25th and 75th percentiles; whiskers extend 1.5 times the interquartile range from the 25th and 75th percentiles; red crosses represent sample means; data points are plotted as gray dots. Letters above boxes refer to individual groups in a one-way ANOVA with a post hoc Tukey test ($p < 0.05$).

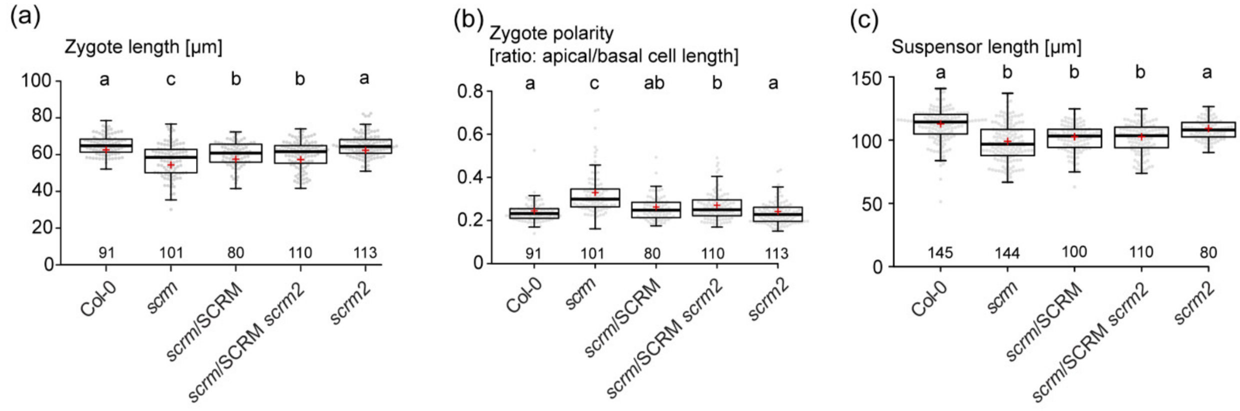

Fig. S4 *gWOX8Δ-YFP* expression is reduced in *scrm* and *wrky2* mutants. Overlay of confocal images of YFP signal (green) and DIC images (gray) of *WOX8* reporter gene expression in zygotes of Col-0, *scrm,* *wrky2*, and *scrm wrky2* double mutants. Scale bar, 10 µm.

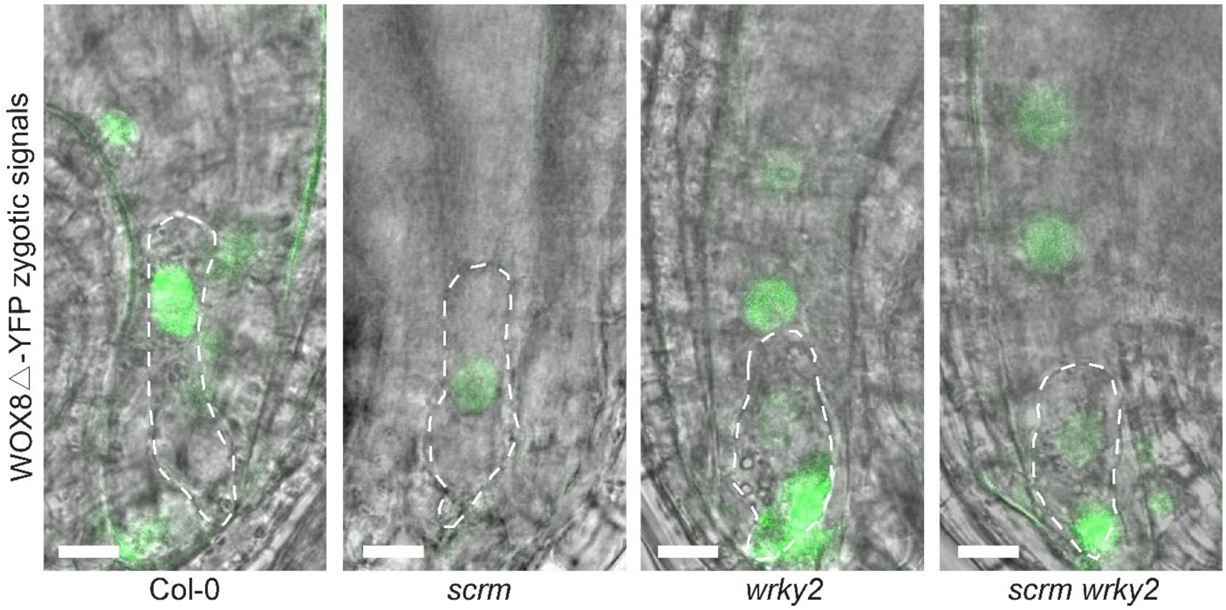

Fig. S5 Stomatal and embryonic phenotypes in transgenic *SCRM* variants. (a) Confocal images of abaxial cotyledon of seedlings 5 days post germination (5dpg). Genotypes are given in the figure panels. Scale bar, 100 µm. (b-e) Boxplot diagrams of phenotype quantifications in *SCRM* variants driven by the *SCRM* promoter. Stomata density (numbers of guard cells are measured in 0.25mm^2 regions); in (b), zygote lengths (c, d) and zygote polarity (e). The sample size is given above the x axis, the genotype is given below. Center lines show the medians; box limits indicate the 25th and 75th percentiles; whiskers extend 1.5 times the interquartile range from the 25th and 75th percentiles; red crosses represent sample means; data points are plotted as gray dots. Letters above boxes refer to individual groups in a one-way ANOVA with a post hoc Tukey test ($p < 0.05$).

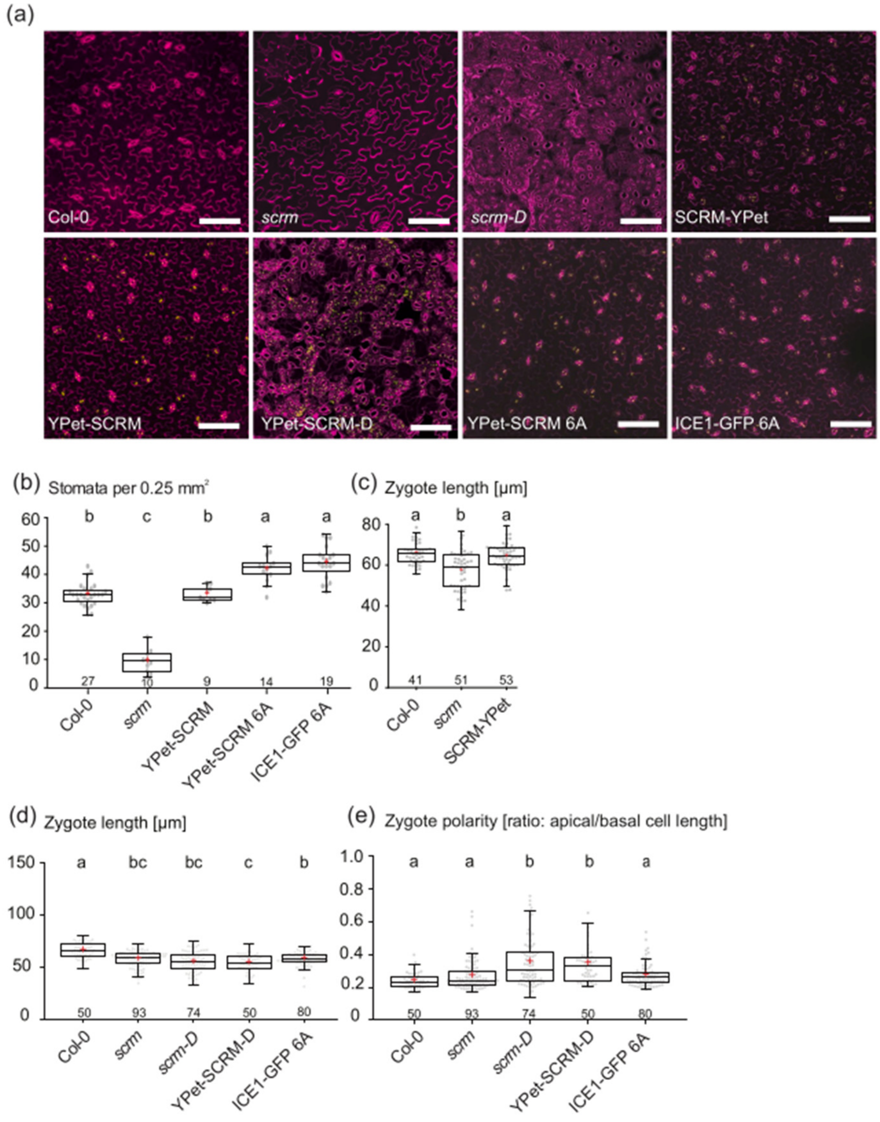

Fig. S6 Gain-of-function defects of constitutively active YDA (*yda-CA*) are partially suppressed in *scrm-D*.

Boxplot diagrams of measured zygote lengths in Col-0, *scrm-D*, *yda-CA*, and *yda-CA scrm-D* double mutant. The *scrm-D* allele interferes with MPK6-directed phosphorylation of SCRM and partially suppresses the effect of *yda-CA* on zygote elongation. The sample size is given above the x axis, the genotype is given below. Center lines show the medians; box limits indicate the 25th and 75th percentiles; whiskers extend 1.5 times the interquartile range from the 25th and 75th percentiles; red crosses represent sample means; data points are plotted as gray dots. Letters above boxes refer to individual groups in a one-way ANOVA with a post hoc Tukey test ($p < 0.05$).

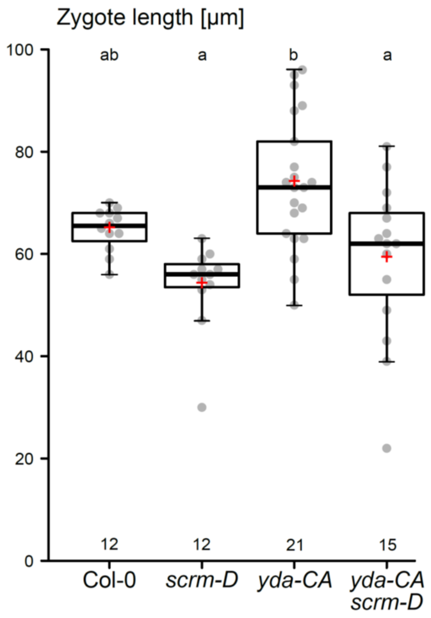

Table S1 Genotyping primer list

Primer Name	Sequences
scrm(SALK_003155)-LP	ATTCTTTGCTCTGCCTCTTCC
scrm(SALK_003155)-RP	TTTGTAGGGCCTTTGTTGTTG
wrky2-1(SALK_020399)-LP	CCAAGAATTTGGCTGAATCTC
wrky2-1(SALK_020399)-RP	TGTTAGAACACGAATCACCCC
scrm2-1(SAIL_808_B10)-LP	TGCATCCGACATCTTTTATCC
scrm2-1(SAIL_808_B10)-RP	TTGTTGTTGTTGCTGTGAAGC
yda-11(SALKseq_078777)-LP	GGGTTGTTGATTAGTAAACCATAT
yda-11(SALKseq_078777)-RP	TAGTAGGAGACCCAGTAGTT
LBb1.3	ATTTTGCCGATTTCGGAAC
SAIL-LB3	TAGCATCTGAATTTCATAACCAATCTCGATACAC
scrm-cr-LP	TATATAAAGACAGAGACAGTGACAACAGAGAC
scrm-cr-MRP	AGTAGGCTGTGCACCAGACGATGCT
scrm-cr-RP	GATCATACCAGCATACCCTGCTGTATC
ssp-6-LP	ATGGTTCTGGTTGGTCCAGGGGA
ssp-6-MLP	TGTTCGAAGAAACGGTGATCGAGC
ssp-6-RP	ACTGGTCTTGGCAAAAGGGAGATTCA

Table S2 Cloning and mutagenesis primer list

Primer Name	Sequences
SCRM-YPet-InF	TAGAACTAGTGGATCCAGACAACCGGACCACCGTCAATAACAT
SCRM-YPet-InR	CACCATGGATCCGCCTCCACCGATCATACCAGCAT
pSCRM(5134)-InF	ATGATTACGAGCTCTCTAGAACTAGTCCTAGGATCTAAAGCCACCTC ATTTGC
pSCRM(5134)-InR	CAACTCCTCACCTTTAGACACCATCGCCAAAGTTGACACCTTTACC
YPet-SCRM-InF	ATGAACGAGTTGTATAAAGGGCCCATGGGTCTTGACGGAAACAATG GTGG
YPet-SCRM-InR	GCCAAGCTGACGTCAGGTACCTAACCGCCATTAACTATGTCTCCTC
SCRM-D-muF	[Phos]-TTCCAGAAACATGCAGCTATGCGTCAGAGCTCT
SCRM-D-muR	[Phos]-GCATAGCTGCATGTTTCTGGAACAGAGTAGGCT
SCRM-S94A-muF	[Phos]-TCTTCTTCTTGTGCTCCTTCTCAAGCTT
SCRM-S94A-muR	AAGCTTGAGAAGGAGCACAAGAAGAAGA
SCRM-S203A-muF	[Phos]-GGAAGGTTTTGGTGCTCCTGCTAATGGT
SCRM-S203A-muR	ACCATTAGCAGGAGCACCAAAACCTTCC
SCRM-T366A-muF	[Phos]-TGAACTTGAGTCAGCTCCTCCTGGATCT

SCRM-T366A-muR	AGATCCAGGAGGAGCTGACTCAAGTTCA
SCRM-T382A T384A-muF	[Phos]-AAGCTTCCATCCGTTGGCACCTGCACCGCAAAC
SCRM-T382A T384A-muR	GTTTGCGGTGCAGGTGCCAACGGATGGAAGCTT
SCRM-S403A-muF	[Phos]-GTTGTGTCCCTCTTCTTTACCAGCTCCTAAAGGCCAGCAA
SCRM-S403A-muR	TTGCTGGCCTTTAGGAGCTGGTAAAGAAGAGGGACACAAC
SCRM-Nluc-InF	GGGACGAGCTCGGTACCATGGGTCTTGACGGAAAC
SCRM-Nluc-InR	GTACGAGATCTGGTCGACGATCATACCAGCATACC
WRKY2-CCluc-InF	CGGGGGACGAGCTCGGTACCATGGCTGGTTTTGATGAAAATGTTGC
WRKY2-CCluc-InR	ACGAGATCTGGTCGACAATCTGAGGTAATCTACTCATGATCTGGTTA TATACC
SPCH-CCluc-InF	CGGGGGACGAGCTCGGTACCATGCAGGAGATAATACCGGATTTTCTT G
SPCH-CCluc-InR	ACGAGATCTGGTCGACGCAGAATGTTTGCTGAATTTGTTGAGC
SCRM-JIT60-InF	CTTGGCTGCAGGTCGACGGATCCATGGGTCTTGACGGAAACAATG
SCRM-JIT60-InR	ATTTCAGCGTACCGAATTCTCAGATCATACCAGCATACCCTGCT
bHLH35-JIT60-InF	TTGGCTGCAGGTCGACATGGAGGATATCGTCGACC
bHLH35-pJIT60-InR	TCAGCGTACCGAATTCTTAGTAAAGAGAGTCGATG
cisA-LUC-InF	AATTCCTGCAGGGATCCATAGTCAAATTAGATTAT
cisA-LUC-InR	GAAGGGTCTTGCACTAGTAATTCCTACTCTTAAAT
35Smini-LUC-InF	CCGGGGGATCCACTAGTGCAAGACCCTTCCTCTAT
35Smini-LUC-InR	TTTGGCGTCTTCCATGGGTCGTCCTCTCCAAATGA
pWOX8-LUC-InF	CGGGGGATCCACTAGTCATTTCCTTGCAAAAACTC
pWOX8-LUC-InR	TTGGCGTCTTCCATGGGATGATGGTGTAATGATGATAATCGAGAGCT

References

Zhao P, Zhou X, Shen K, Liu Z, Cheng T, Liu D, Cheng Y, Peng X, Sun M-X. 2019. Two-step maternal-to-zygotic transition with two-phase parental genome contributions. *Developmental Cell* **49**(6): 882-893. e885.
